# Research on Selected Properties of Concrete Composite with the Addition of Post-Production Metallic Dust

**DOI:** 10.3390/ma18225197

**Published:** 2025-11-15

**Authors:** Bogdan Langier, Izabela Major

**Affiliations:** Faculty of Civil Engineering, Czestochowa University of Technology, 69 Dąbrowskiego Street, 42-201 Częstochowa, Poland; bogdan.langier@pcz.pl

**Keywords:** metallic dust, concrete composite, compressive strength, abrasion resistance, frost resistance

## Abstract

A major issue in industrial production is the generation of post-production wastes that are not biodegradable. The article presents an innovative solution for the management of industrial waste, which includes, among others, metal dust generated during the grinding of castings. The results of research on a concrete composite modified with metallic dust, a by-product from cast iron product manufacturing, were presented. The study analyzed the effect of using metallic dust as a partial replacement for fine aggregate at levels of 10%, 20%, 30%, 40%, and 50% on selected concrete properties. Tests included concrete mix consistency, compressive strength after 28 days and 6 months, density after 28 days of curing, bending strength, abrasion resistance using the Boehme disk method, durability in a salt chamber, and air content in hardened concrete. The research results indicate the possibility of using waste metal dust in concrete composites as a substitute for sand as a fine aggregate. An innovative waste processing solution allows the creation of a product with better abrasion resistance and compressive strength parameters while also having a good impact on the environment.

## 1. Introduction

The increase in industrial production results in the generation of ever-growing amounts of waste. The search for optimal waste management methods aligns with the broader trend of implementing the principles of the circular economy, in which recycling plays a fundamental role. The sustainable use of natural resources has made the reuse of post-production waste a global trend in waste disposal strategies. Numerous studies from various research centers have addressed the application of waste materials in concrete technology [1,2,3]. Concrete is currently the most widely used construction material worldwide. A key direction in the recycling of various types of waste—such as polyethylene terephthalate (PET) [4,5], ceramics [6], crushed side window glass [7], fly ash [8], waste tire rubber [9], metallurgical industry by-products [10], and others—is their use as aggregate replacements in concrete [11].

Natural aggregates are the main component of concrete, accounting for more than 70% of its volume [12], and their global annual consumption is estimated at approximately 20 billion tons [13]. Substituting aggregates with industrial waste may significantly reduce natural aggregate extraction and mitigate environmental degradation [14,15].

Many publications present the use of fired red clay ceramic waste [16] and sanitary ceramic waste [17,18,19] as partial aggregate substitutes. The findings of these studies show that concretes with ceramic fillers exhibit mechanical properties comparable to conventional concretes, and that the production process of concrete mixtures does not require modification. In addition, aggregates from recycling processes—e.g., from the reconstruction and expansion project of the expressway—are also used in concrete production. In the paper [20], the authors used aggregates processed from construction waste, including concrete, mortar, stone, and bricks, with particle sizes ranging from greater than 4.75 mm to less than 31.5 mm, conforming to a continuous gradation of 5–31.5 mm. In the study [21], the authors focused on exploring the potential of utilizing demolished concrete through the use of recycled concrete aggregate (RCA) as a substitute for natural aggregates.

The use of steel fibers and steel chip waste as reinforcement in concrete also supports the principles of sustainable development. The addition of steel fibers in appropriate amounts can significantly improve the structural performance and strength of concrete [22]. Reusing industrial waste, such as steel chips, as recycled aggregate improves compressive strength and stiffness, while also reducing the environmental impact of concrete [23,24].

Metallurgical by-products are classified as hazardous waste due to their potential environmental impact [11]. During surface treatment processes of cast iron products—such as grinding or polishing—significant quantities of fine metallic waste are generated. These are classified under Group 12 in the Polish Waste Catalogue, as defined by the Regulation of the Minister of Climate [25]. The largest share of waste from the foundry industry is constituted by waste from molding and core sands, the amount of which is estimated at about 80% of the total amount of waste generated in metal foundries. The waste generated during the production of castings also includes dust, chips, and abrasive materials, which can constitute up to 10% of the waste [26].

Their reuse, for example, in concrete technology, is consistent with the idea of sustainable development. The basic component of dust from grinding castings is the material itself that was processed, and the abrasive material used in the grinding process. Waste from the surface treatment of cast iron products could serve as a substitute for natural mineral resources, significantly reducing the extraction of natural raw materials [27].

Steel dust is a fine substance that can be used in place of fine aggregate, primarily due to comparable particle sizes. In the paper [28], the authors investigated the effect of steel chip waste from wheel flanges used as an aggregate substitute. The impact of adding wasted steel dust on the mechanical behavior of concrete was studied by partially replacing sand in various percentages (0%, 3%, 6%, 9%, 12%, and 15%). Adding steel dust to concrete generally increases its strength. Optimal values for compressive and flexural strength were observed in concrete containing 9% steel dust, while the highest tensile strength was recorded in concrete containing 15% steel dust. In the study [29], the mechanical properties of concrete were studied using metal aggregate from blacksmith forging waste. The metal aggregates were used to replace sand as the fine component in the concrete mix. The test results showed an improvement of nearly 17% in compressive strength. In the study [30], an analysis was conducted on the effects of integrating steel dust as a partial substitute for cement in reinforced concrete beams. The results showed an increase in compressive strength with a 10% cement replacement, but a decrease at higher replacement levels. The inclusion of 10% steel dust increased ductility, whereas a 30% inclusion reduced both ductility and the maximum load capacity. The mechanical behavior of concrete samples incorporating industrial waste, such as ferritic dust generated by electric arc furnaces (EAFs), was also investigated [31]. The results demonstrated that, in addition to enhancing the durability of concrete, the use of such waste is environmentally viable. The waste remains encapsulated within the concrete matrix, preventing the leaching of heavy metals that could be harmful to the environment and, consequently, to human health.

In this work, the developed concrete mix recipe was modified with waste dust from grinding castings made of GJL-200 gray cast iron. These dusts were used as a partial replacement for fine aggregate in amounts ranging from 10% to 50% of the mixture volume. The aim of the research was not only to determine the impact of this type of waste on the physical and mechanical properties of concrete, but also to develop a solution consistent with the principles of sustainable development and the circular economy, the implementation of which is currently one of the priorities of European Union policy.

The use of waste dust in concrete mixtures can be considered as an action consistent with the idea of sustainable construction, which aims to minimize the negative impact of construction investments on the environment, while maintaining or improving the quality and durability of the produced materials. From the perspective of a circular economy, such initiatives constitute an important step towards a fuller use of the potential of waste materials and the development of innovative, low-emission technologies in construction.

The results of tests on concretes that can be used in the construction of industrial floors exposed to intense abrasion and therefore requiring high mechanical resistance and durability are presented. The use of waste dust in this type of concrete not only allows for the improvement in the analyzed performance parameters, but also constitutes an element of the strategy of sustainable design of building materials, which aims to limit the consumption of primary mineral raw materials and minimize the amount of industrial waste.

The introduction of this type of waste material into concrete formulations is an example of the practical implementation of the assumptions of the European Green Deal and the 2030 Agenda for Sustainable Development, in particular Goal 12: Responsible Consumption and Production. The use of recycled raw materials in concrete technology contributes to reducing the carbon footprint of the production process, limiting the amount of waste sent to landfills, and also reducing the pressure on the environment resulting from the exploitation of natural aggregate deposits.

The use of waste dust from grinding castings as a fine aggregate substitute also has economic justification. It is more cost-effective for casting manufacturers to provide this waste material free of charge than to incur the costs of its disposal [32].

The literature review conducted showed that previous research has focused mainly on the use of metallic waste, such as metallurgical slag, fly ash, or dust from metallurgical processes, but there are no studies on the direct use of waste dust from grinding castings as a component of concrete mixtures. Therefore, this research is an attempt to fill the existing research gap by indicating the possibility of effective management of waste that has so far been treated as difficult to reuse.

The thesis that adding fine filler in the form of dust from the surface treatment of castings can improve the microstructure of concrete by reducing its porosity, improving the adhesion between aggregate grains and cement paste, and increasing the structural density of the composite was proposed. The effect of these changes is a potential increase in concrete’s resistance to abrasive wear, which is important in the context of the durability of structural elements used in industry.

## 2. Purpose and Scope of the Research

The presented literature review indicates that, at present, there is no information on the use of waste from the surface treatment of cast iron products, in particular gray cast iron EN-GJL-200, in concrete technology. The research program within this work included the design of seven variants of concrete mixtures to analyze the effect of the amount of metal dust waste on selected properties of modified concretes:

- Series 1 (K0)—reference concrete with superplasticizer;
- Series 2 (KF)—a variant in which Series 1 has been modified with the addition of dispersed reinforcement in the form of steel fibers;
- Series 3–7 (KM) variants, in which Series 1 was modified with the addition of metallic dust, used as a partial replacement for fine aggregate in amounts of 10, 20, 30, 40, and 50% (5 series KM1 to KM5).

Experimental tests of concrete mixtures and hardened concrete included the following:

- Testing the consistency of concrete mixtures;
- Marking the density of concrete composites;
- Testing the abrasion resistance;
- Evaluation of compressive and bending strength;
- Frost resistance assessment;
- Salt chamber test;
- Testing for air content in hardened concrete.

## 3. Composition of Tested Concrete Composites

**Figure 1:**
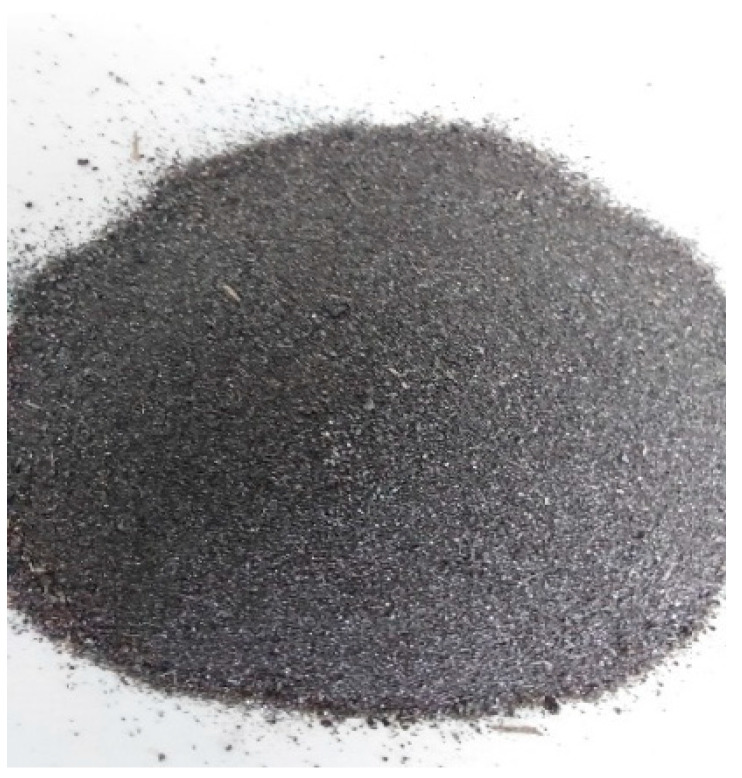
Metallic dust used in the study.

The metallic dust used in this study (Figure 1) was analyzed for chemical composition using the SPECTRO XEPOS XRF fluorescence spectrometer (Rigaku EDXRF, Tokyo, Japan) with energy dispersive X-ray (ED-XRF). The results are shown in Table 1.

The highest content in the tested additive is iron compounds. The spectral analysis of the used GJL-200 gray cast iron is summarized in Table 2.

GJL-200 is a low-quality gray cast iron characterized by good machinability, abrasion resistance, and vibration damping, as well as good thermal conductivity. It has a tensile strength of approximately 200 MPa and is commonly used in the production of machine parts, housings, manhole covers, and drainage pipes. The carbon in this alloy is in the form of flake graphite, which gives it brittleness but also good damping properties.

**Figure 2:**
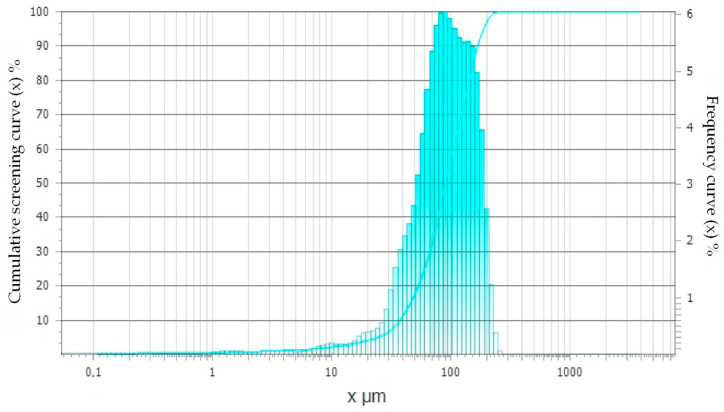
Graphical representation of the particle size distribution of metal dust.

The grain size distribution of metal dust was also determined using the Analysette 22 laser particle size analyzer (Sigma-Aldrich, St. Louis, MO, USA). The obtained results are presented in Figure 2, while the grain size distribution of metal dust is presented in Table 3.

The analysis showed that 50% of particles were below 90.79 µm, and 90% were below 170.11 µm. The tests of the additive effect were carried out on concrete composites made of basalt aggregate with the particle size presented in Table 4.

To make the concrete composites, Portland cement CEM II/B-M (S-V) 42.5N (Cemex, Rudniki, Poland) and superplasticizer Chryso^®^Optima 294 (Chryso (part of Saint-Gobain Construction Chemicals), Błonie, Poland) were used.

**Figure 3:**
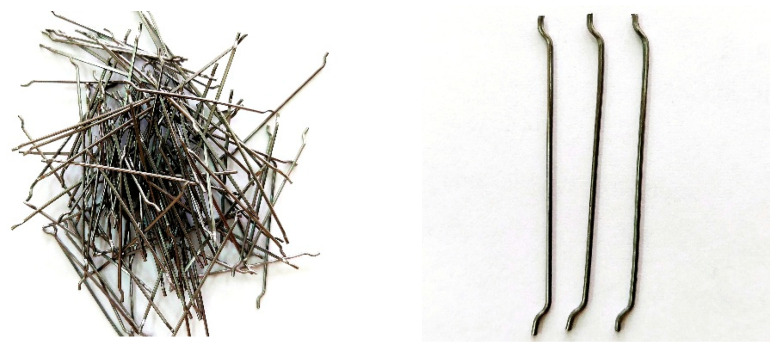
Steel fiber used in the KF series.

The tests were performed for seven different series of concrete composites. The control series K0 was modified with the addition of metal dust used as a partial substitute for fine aggregate in amounts of 10, 20, 30, 40, and 50% (5 series KM1 to KM5), and an additional series was made with the addition of dispersed reinforcement in the form of steel fibers (Figure 3) (series KF); the composition of the control series K0 and the introduced modifications are presented in Table 5. Steel fibers of 50 mm length and 1 mm diameter with hooks at both ends were used, certified for structural use according to [33].

The KM1–KM5 series, incorporating metallic dust as a partial replacement for fine aggregate in amounts from 10% to 50%, was designed incrementally to investigate the effect of increasing waste content on concrete properties. The selection of replacement levels was informed by prior studies on metal dust and other fine industrial by-products used in concrete, which indicated that partial replacement within a similar range can enhance compressive strength, bending strength, abrasion resistance, and durability without adversely affecting workability [28,29,30,31].

The adjustment of the superplasticizer dosage in the KM series was necessary to maintain a consistent slump (S3 class) across all mixes.

The gradual replacement of sand with metallic dust while keeping the total aggregate mass constant ensures a controlled assessment of the impact of the additive on the concrete’s physical and mechanical properties.

## 4. Research Methodology

The assessment of the consistency of the concrete mixture was carried out using the slump test according to [34]. The method involves placing and compacting the concrete mixture in a form in the shape of a truncated cone. The slump of the concrete mix cone (after removing the form) is a measure of its consistency. The obtained result allows the concrete mix to be classified into one of the consistency classes (Table 6).

The bulk density of concrete composites was tested in accordance with [36]. The determination was performed for three cubic samples with nominal dimensions of 100 mm × 100 mm × 100 mm.

The determination of abrasion resistance was performed according to the procedure of [37]. Samples left after density determination were used for the study. From each series, three samples measuring 70 mm × 70 mm × 70 mm were prepared for abrasion testing on the Boheme disk.

The samples were subjected to 16 grinding cycles, each with 22 disk revolutions. The abrasion resistance was determined as the average reduction in the sample volume Δ*V* based on the mass loss Δ*m* after 16 cycles according to the formula:

(1) $$∆V=\frac{{∆}_{m}}{\rho_{b}}\;[mm^{3}],$$

where $\rho_{b}$ is the bulk density.

The compressive strength test was performed according to [38]. Compressive strength tests after 28 days of curing were performed on five cubic samples of 150 mm × 150 mm × 150 mm for each series of concrete composites.

The bending strength tests were carried out according to [39] on three beams of dimensions 400 mm × 100 mm × 100 mm.

The frost resistance test was carried out after 90 days of curing of concrete composites using the conventional method for 50 freeze/thaw cycles according to [40]. Twelve cubic samples with an edge dimension of 100 mm were prepared for each series. Six of the twelve samples were left in water (control samples), while frost resistance was determined on the remaining six samples. After the last cycle, the samples were weighed, and their compressive strength was tested.

The evaluation of the behavior of concrete composites in a corrosive environment was performed using a salt chamber test in a sprayed 5% sodium chloride NaCl solution at a temperature of 35 °C for 48 h. The test was performed on samples with dimensions of 100 mm × 100 mm × 50 mm, obtained from cutting out samples after the bending strength test.

The porosity of hardened concrete was also tested by determining the characteristics of the air voids distribution in hardened concrete in accordance with the standard [41]. The porosity structure was examined using an image analysis device and the Lucia Concrete computer program.

## 5. Research Results

### 5.1. Consistency of Concrete Mixtures

The results of testing the consistency of concrete mixtures using the slump cone method are presented in Table 7.

Metallic dust, due to its water demand, caused a decrease in the fluidity of the concrete mix. In order to maintain a constant consistency of the tested mixtures, an increased amount of superplasticizer was introduced. All tested series of concrete mixes obtained a degree of fluidity expressed by the consistency class S3. Increasing the superplasticizer dose translates into an increase in the effective amount of water in the mixture recipe, which translates into an increase in the water/cement ratio W/C. A higher W/C ratio may result in a decrease in compressive strength.

### 5.2. Marking the Density of Concrete Composites

The obtained result of the average value of the tests is presented in Table 8.

Due to the higher volumetric weight of the additive used in the modified series, an increase in the volumetric density of concrete composites is clearly visible.

### 5.3. Abrasion Resistance

The results of determining the abrasion resistance on the Boheme disk are presented in Table 9.

For the K0 series, a volume loss of 14.9 mm^3^ was measured; all other series achieved a smaller loss. In the KF series reinforced with steel fiber, the most favorable result was achieved at 9.77 mm^3^, which is a 34% decrease compared to the K0 series. The use of metal dust also influenced the volume loss after the test, which decreased with the increase in the amount of additive. The best result among the dust series was obtained in the KM4 series, which contained 40% of grinding waste.

### 5.4. Evaluation of Compressive and Bending Strength After 28 Days of Curing

**Figure 4:**
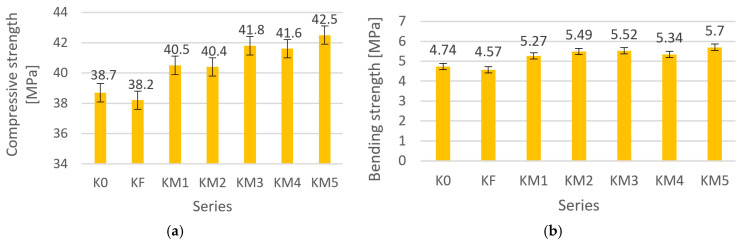
Average (**a**) compressive and (**b**) bending strength of the tested concrete composites.

The test was carried out on five cubic samples with dimensions of 150 mm × 150 mm × 150 mm for each series of concrete composites. The obtained average value of compressive strength is shown in Figure 4a. The bending strength tests were carried out according to [39] on three beams with dimensions of 400 mm × 100 mm × 100 mm, and the obtained average value is shown in Figure 4b. The results of the strength test in relation to the reference sample K0 are presented in Table 10.

For the K0 control series, the compressive strength was 38.7 MPa, which allows it to be classified as C25/30, similar to the KF series with the addition of steel fiber. The use of metal dust added improved the strength; strengths above 40 MPa were obtained in all tested series, which allows them to be classified as strength class C30/35. The highest strength was obtained for the KM5 series, and it amounted to 42.5 MPa, which is an increase of about 9.8% compared to the K0 series. Although the effective water/cement ratio increased with higher dust content, a beneficial effect of wastes on compressive strength could be observed. The metal dust used in the composition also improved the bending strength.

The compressive strength of the tested concrete composites after 6 months of curing was determined on three cubic samples with a side dimension of 150 mm. The obtained results are presented in Table 11.

In all series, there was an increase in compressive strength over time of approximately 20%. This proves that the additive used did not negatively affect the increase in strength over time, and such a significant increase may be primarily due to the type of cement used, CEM II/B-M (S-V) 42.5N (Cemex, Rudniki, Poland).

### 5.5. Frost Resistance Assessment

**Figure 5:**
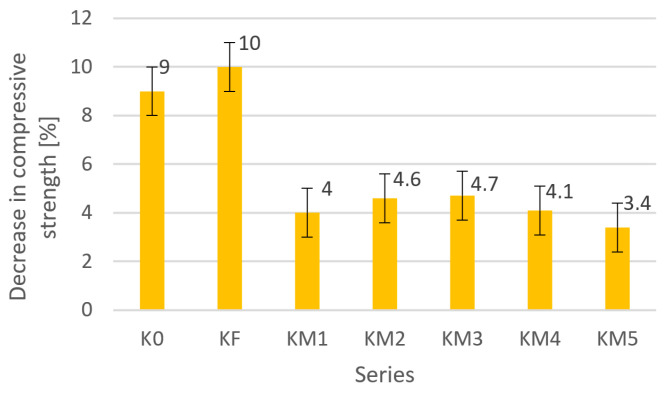
Decrease in compressive strength of tested samples after 50 cycles.

In the assessment of frost resistance after 50 freeze/thaw cycles, after the last cycle, the samples were weighed, and their compressive strength was tested. After the frost resistance test, no loss in the mass of the samples was observed in all the tested series, while the obtained decrease in compressive strength compared to the strength of non-frozen samples for all the tested concrete series is shown in Figure 5.

The reference series K0 achieved a decrease in compressive strength of 9%, similar to the series with the addition of steel fiber KF, for which the decrease was 10%. In all series modified with the addition of metallic dust, the frost resistance of concrete composites improved, and the decrease in compressive strength after the frost resistance test ranged within 4%. The smallest decrease in strength was observed in the KM5 series, in which 50% of the fine aggregate was replaced by the addition of metal dust, and it amounted to 3.4%.

### 5.6. Salt Chamber Test

**Figure 6:**
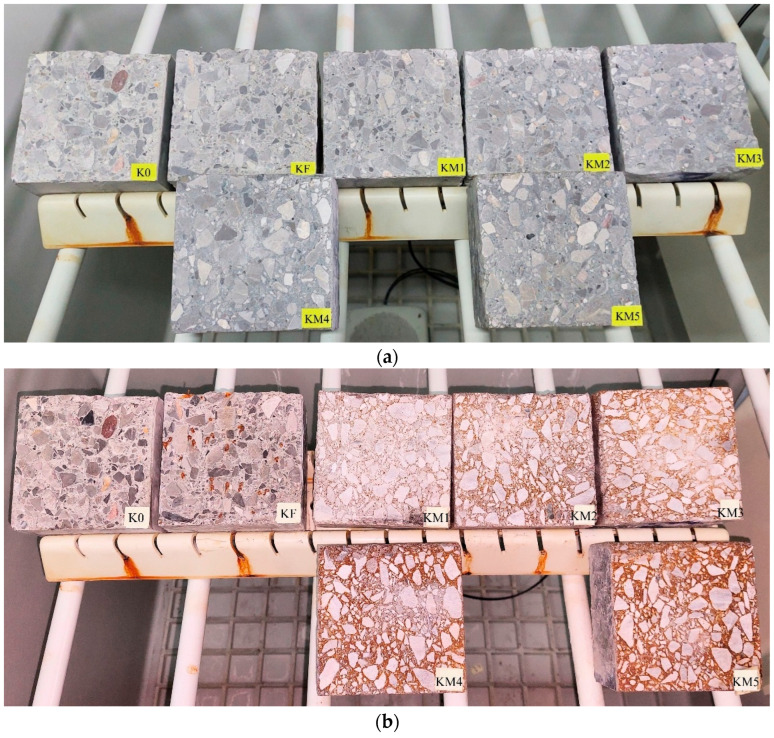
Samples of concrete composites (**a**) before, and (**b**) after testing in a salt chamber.

**Figure 7:**
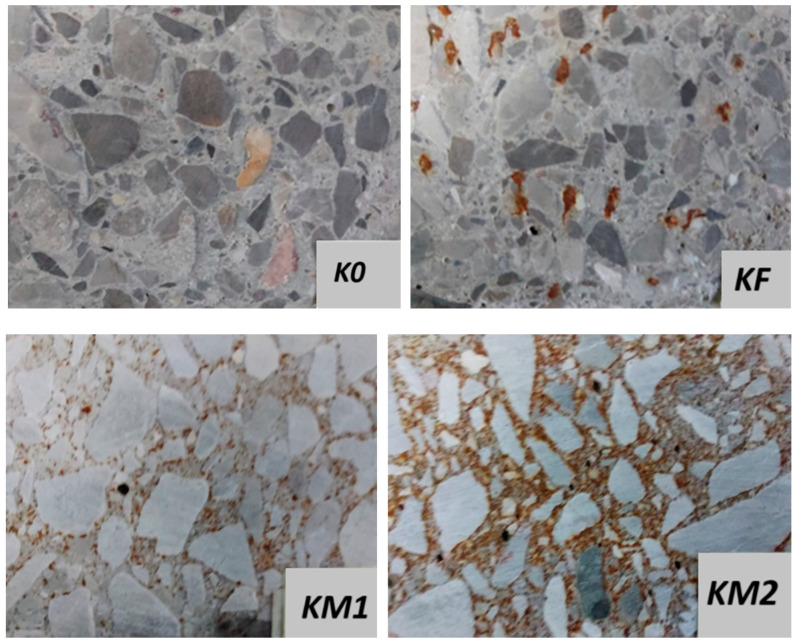

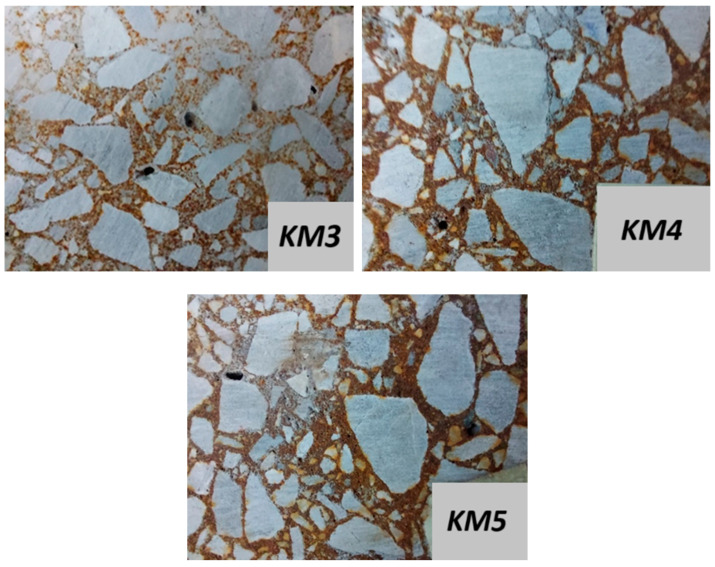
Close-ups of the sample surfaces after testing in a salt chamber.

Salt spray chamber test in a sprayed 5% sodium chloride solution, NaCl, was carried out on samples with dimensions of 100 mm × 100 mm × 50 mm, obtained from cutting out samples after the bending strength test. Photos of the samples before and after the salt chamber test are shown in Figure 6 and Figure 7.

On the surfaces of the samples after testing, traces of corrosion of the metal dusC used in the composites, as well as corrosion and location of the steel fiber in the KF series, are clearly visible. It can be observed that with the increased content of added metal dust, their clear densification in the cement matrix is visible, especially in the contact layer between the matrix and the coarse aggregate grains.

**Figure 8:**
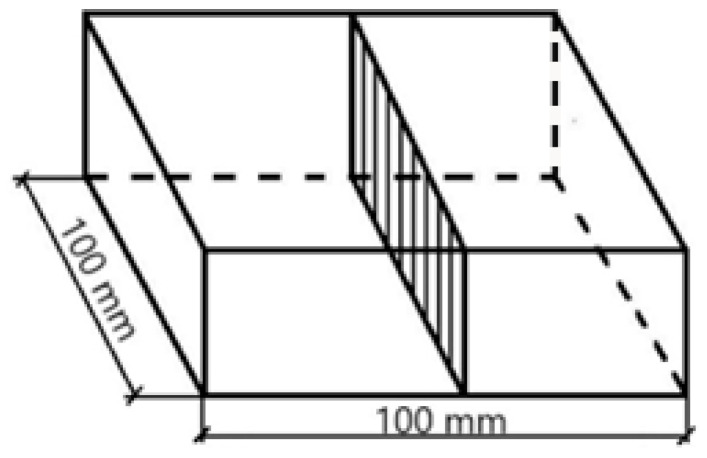
Cutting plane of research samples.

After 60 days from the salt chamber test, the test samples were cut (as shown in Figure 8) to observe any possible corrosion inside the samples. No traces of corrosion were found inside the samples; only surface corrosion occurred.

**Figure 9:**
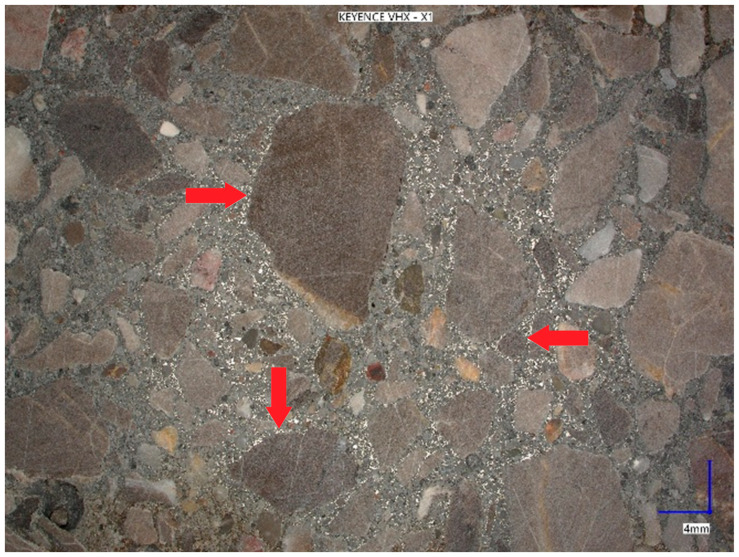
The surface of the sample after cutting, as shown in Figure 8, at five times magnification. (Red arrows indicate the location of the metal dust).

Figure 9 shows a photo of the surface of a KM4 series sample taken with a digital microscope at a fivefold magnification. The concentration of added metal dust in the contact layer between the matrix and the aggregate grains is clearly visible, which results in the sealing of the concrete composite. Sealing the contact zone between the grout and the aggregate grains significantly improved the mechanical properties, as well as the frost resistance of the concrete.

**Figure 10:**
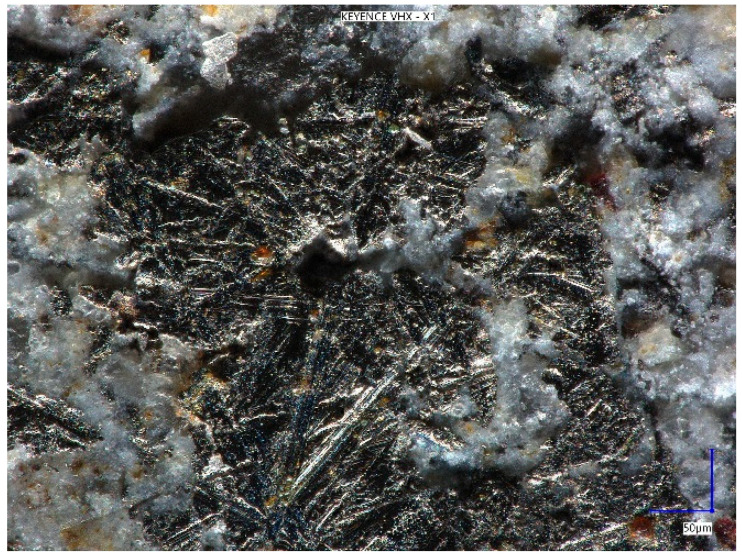
Metal dust inclusions (×900).

The surface of fine grains of the used metal dust is expanded (Figure 10), which improves adhesion to the cement paste.

**Figure 11:**
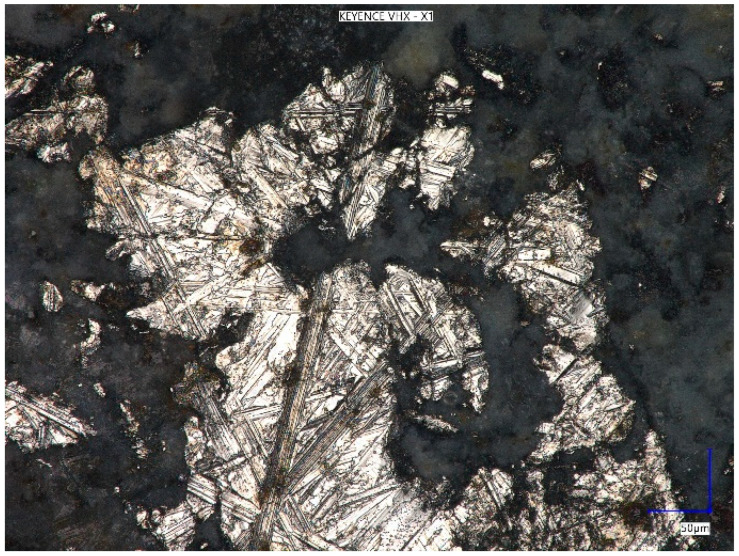
Surface of metal dust at 900× magnification.

**Figure 12:**
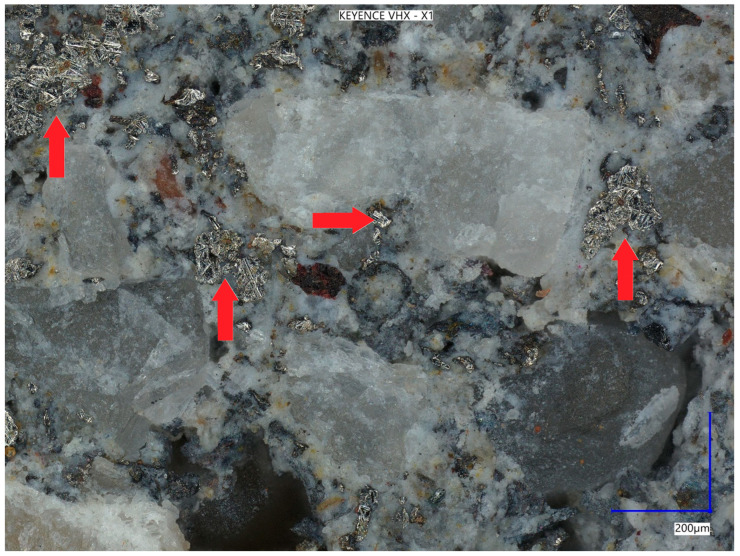
Sample surface at 350× magnification. (Red arrows indicate the location of the metal) dust.

Figure 11 shows a photo of a metal dust grain in the composition of the concrete composite taken at 900× magnification, while Figure 12 shows the surface of the sample at 350× magnification.

### 5.7. Testing for Air Content in Hardened Concrete

To assess the tightness of modified concretes, a test according to the standard [41] was used. This method allows the determination of the total air content in hardened concrete A [%], and the test results are presented in Table 12.

The obtained result of the test of air content in hardened concrete indicates a clear sealing of the concrete structure with the increasing amount of waste dust from casting processing used. The control series K0 had an air content of 3.0%, and the lowest amount was recorded in the series KM5, containing 50% waste. This confirms the achievement of tighter concrete resulting from the sealing of the aggregate–grout contact zone, which is visible in the structure photos (Figure 9).

## 6. Discussion

In the test of the consistency of concrete mixtures, a worsening degree of fluidity was observed with the increased amount of metal dust added. In order to maintain a constant or similar consistency in all series, it was necessary to use an increased dose of superplasticizer. This treatment increases the effective water/cement ratio in the concrete mix.

Compressive strength tests have shown that the use of industrial waste in the form of metal dust in the composition of the tested concretes improves this property. An increase in compressive strength was demonstrated with an increased amount of metal dust despite the increased effective water/cement W/C ratio.

In the KM5 series with the highest W/C ratio and the highest dust content, the compressive strength increased by almost 10% compared to the reference K0 series. The K0 series achieved a concrete strength class of C25/30, while the KM30 series, through the KM40 and KM50 series, achieved a higher concrete strength class of C30/37. This proves the significant influence of the additive used on the compressive strength. The use of dispersed reinforcement in the form of metallic dust proved to be a more advantageous solution than steel fiber reinforcement.

In the assessment of frost resistance of the tested composites after 50 cycles, a lower decrease in compressive strength was found after the test in all series containing dust waste than in the reference series K0. This result indicates an improvement in the frost resistance of the composites and is a more favorable behavior than for steel fiber reinforcement, which did not improve the frost resistance of the composite. This result is due to the sealing of the composite structure, which was confirmed by tests of the total air content in concrete.

In the assessment of abrasion resistance, an improvement in this feature was achieved in all series modified with the addition of metallic dust precipitation. The abrasion resistance of the modified series was comparable to the abrasion resistance of the series reinforced with steel fiber. The dispersed reinforcement allowed for a volume reduction of 34% compared to the control series, and the KM5 series improved this feature by approximately 27%. This proves that the metal dusts used in grinding castings can be successfully used in cement composites exposed to abrasion during use.

In interpreting the obtained results, several assumptions and limitations of the study should be acknowledged. The metallic dust used in the research was assumed to be chemically stable and representative of typical cast iron grinding residues, without significant oxidation or variation in composition during mixing and curing. The particle size distribution and morphology of the metallic dust were considered constant throughout the experimental program, ensuring the comparability of results.

The main limitations of this work arise from the laboratory scale and controlled environmental conditions under which the tests were performed. Therefore, the outcomes may not fully reflect the variability observed in industrial-scale concrete production.

## 7. Conclusions

The obtained research results allow us to formulate the following conclusions:

- Concrete composites with the addition of metal dust require an increased amount of a fluidifying admixture in order to maintain the fluidity of the concrete mixture due to their higher water demand;
- The used fine aggregate substitute had a significant effect on the abrasion of concrete composites, reducing the volume loss in the KM4 series by up to 29% compared to the reference K0 series, which indicates their potential use as reinforcement of industrial floors, contributing to improving the quality of the surface and extending its service life including protection, e.g., with a hydrophobic impregnation that penetrates deep into the concrete, thus providing effective protection against the penetration of contaminants and possible corrosion;
- Metal dust in the tested concrete composites in each case improved the bending and compressive strength, and for the KM5 series, the increase in compressive strength was about 10% compared to the reference K0 series;
- In the study of changes in compressive strength over a period of up to 6 months, no negative impact of the metal dust used was found;
- The used fine aggregate substitute improved the frost resistance of concrete after 50 freezing and thawing cycles. In each case of modification of the concrete composite with metal dust, the decrease in compressive strength after the test was lower than in the K0 control series. The KM5 series achieved a decrease in compressive strength lower by more than 50% compared to the K0 series;
- The process of processing waste materials into raw materials brings benefits in the form of reduced consumption of natural resources and, at the same time, contributes to reducing the costs of transporting waste to landfills, which extends the service life of the landfill;
- Statistical validation of the obtained results was performed using one-way ANOVA to evaluate the significance of differences between the reference series (K0) and modified concretes (KM1–KM5). The analysis confirmed that the addition of metallic dust caused statistically significant improvements in compressive and flexural strength and a reduction in abrasion loss. Therefore, the observed enhancement in performance parameters is statistically supported.

Future prospects:

- Further studies are needed to evaluate long-term durability under real environmental conditions, including exposure to aggressive chemical agents and variable climates;
- Optimization of mixture design and exploration of combined use with other industrial by-products could further enhance the mechanical and durability properties of sustainable concrete composites;
- The practical implementation of metallic dust in structural and industrial concrete offers a promising strategy for eco-friendly construction and industrial waste management.

## Figures and Tables

**Table 1:** Chemical composition of metallic dust.

Iron(III) Oxide Fe_2_O_3_	Silicon Dioxide SiO_2_	Aluminum Oxide Al_2_O_3_	Sulfur Trioxide SO_3_	Phosphorus Pentoxide P_2_O_5_	Manganese Mn	Magnesium Oxide MgO	Calcium Oxide Ca
88.51	7.979	2.176	0.5852	0.471	0.532	0.196	0.1188

**Table 2:** Spectral analysis of gray cast iron GJL-200.

C	Si	Mn	P	S	Cr	Ni	Mo	Mg
%								
3.34	2.32	0.53	0.031	0.014	0.052	0.024	0.017	0.009

**Table 3:** Particle size distribution of metallic dust.

%	10	50	90
<µm	37.46	90.79	170.11

**Table 4:** Percentage content of aggregate fractions.

Fraction [mm]	Contents [%]
0–0.063	0.5
0.063–0.125	2.5
0.125–0.25	10
0.25–0.5	14
0.5–1.0	7
1.0–2.0	5
2.0–4.0	13
4.0–8.0	18
8.0–16.0	30

**Table 5:** Composition of the tested concrete composites per 1 m^3^ of the mixture.

Series	K0	KF	KM1	KM2	KM3	KM4	KM5
	[kg]						
Cement	290						
Water	174						
Superplasticizer	3.77	3.77	4.35	4.35	5.8	5.8	7.25
Sand	677	677	609.3	541.6	473.9	406.2	338.5
Basalt 4–16 mm	1303						
Metallic dust	--	--	67.7	135.4	203.1	270.8	338.5
Aggregate Ʃ	1980						
Steel fiber	--	30	--	--	--	--	--

**Table 6:** Consistency classes determined by the cone slump method [35].

Class	Slump [mm]
S1	10–40
S2	50–90
S3	100–150
S4	160–210
S5	>210

**Table 7:** Consistency of the mixture of tested concrete composites.

Series	K0	KF	KM1	KM2	KM3	KM4	KM5
Slump test [mm]	140	120	115	110	100	120	115
Consistency class	S3	S3	S3	S3	S3	S3	S3

**Table 8:** Bulk density test results for concrete composites.

Bulk Density [kg/dm^3^]						
K0	KF	KM1	KM2	KM3	KM4	KM5
2.385	2.426	2.444	2.461	2.488	2.507	2.557

**Table 9:** The results of the abrasion test on the Boheme disk.

Abrasion-Volume Loss [mm^3^]						
K0	KF	KM1	KM2	KM3	KM4	KM5
14.9	9.77	11.7	11.3	11.1	10.5	10.9
---	↓34%	↓21.5%	↓24%	↓25.5%	↓29.5%	↓26.8%

↓ indicates a decrease in value compared to the K0 series.

**Table 10:** The results of the strength test compared to the reference sample K0.

Series	K0	KF	KM1	KM2	KM3	KM4	KM5
*f_cm_*—compressive strength [MPa]	38.7	38.2	40.5	40.4	41.8	41.6	42.5
Standard deviation	0.70	0.89	0.63	0.47	0.85	0.31	0.63
Coefficient of variation	0.018	0.023	0.016	0.012	0.020	0.007	0.015
Percentage change with respect to K0 [%]	----	↓1.3	↑4.7	↑4.4	↑8.0	↑7.5	↑9.8
*f_zg_*—bending strength [MPa]	4.74	4.57	5.27	5.49	5.52	5.34	5.7
Standard deviation	0.14	0.147	0.487	0.369	0.124	0.222	0.483
Coefficient of variation	0.029	0.032	0.092	0.067	0.022	0.042	0.085
Percentage change with respect to K0 [%]	----	↓3.6	↑11.2	↑15.8	↑16.5	↑12.7	↑20.2

↓ indicates a decrease in value compared to the K0 series. ↑ indicates an increase in value compared to the K0 series.

**Table 11:** Compressive strength test results after 6 months.

Compressive Strength After 6 Months [MPa]							
Series	K0	KF	KM1	KM2	KM3	KM4	KM5
*f_cm_*—compressive strength [MPa]	46.0	45.8	48.7	49.2	50.3	49.5	49.7
Standard deviation	0.32	0.35	0.71	0.56	0.50	0.67	0.55
Coefficient of variation	0.007	0.008	0.015	0.011	0.010	0.013	0.011
Percentage change [%]	18.9	19.7	20.4	21.7	20.4	19.0	17.0

**Table 12:** Test results of the characteristics of the distribution of air voids in concrete.

Series	K0	KF	KM1	KM2	KM3	KM4	KM5
Total air content in concrete A [%]	3.0	3.2	2.9	2.8	2.6	2.4	2.3

## Data Availability

The original contributions presented in the study are included in the article, further inquiries can be directed to the corresponding author.
